# Toward high-stability quantitative carriers: development of superparamagnetic nanocarriers and a high-sensitivity mixing-frequency narrow-band magnetic particle imaging detection system

**DOI:** 10.3389/fphar.2026.1773881

**Published:** 2026-05-08

**Authors:** Tianshu Li, Yichao Wang, Xin Zheng, Huiyan Si, Yue Kang, Hongye Chen, Shi Bai

**Affiliations:** 1 Department of Information Science and Technology, Shenyang University of Technology, Shenyang, Liaoning, China; 2 Department of General Surgery, The First Medical Center, Chinese PLA General Hospital, Beijing, China; 3 Department of Breast Surgery, Cancer Hospital of Dalian University of Technology, Cancer Hospital of China Medical University, Liaoning Cancer Hospital & Institute, Shenyang, Liaoning, China; 4 Department of General Surgery, The 8th Medical Center, Chinese PLA General Hospital, Beijing, China

**Keywords:** magnetic particle imaging, mixing-frequency, nanocarrier, narrow-band, quantifiable fluctuation, quantitative imaging

## Abstract

**Introduction:**

Superparamagnetic iron oxide nanoparticles (SPIONs) have attracted extensive attention in recent years as visualizable and controllable organic nanocarriers. Magnetic particle imaging (MPI), as a quantitative imaging method, enables visual and quantitative characterization of carriers. The quantitative imaging capability of carriers is critically crucial for precise quantitative local drug concentrations. However, the alternating current susceptibility (ACS) of SPIONs is highly susceptible to external interference and, thus, prone to fluctuations, which tend to be multifactorial and poorly defined in the *in vivo* environment, posing a major challenge to the quantitative analysis of carriers in living organisms.

**Methods:**

Through the analysis of the third-harmonic ACS of SPIONs, we proposed that under specific nanoscale dimensions and instrument frequency, the ACS maintained a relatively stable state, with a quantifiable fluctuation range even under complex interference conditions, thus enabling quantitative analysis in the *in vivo* environment. Meanwhile, to achieve precise detection of carriers with signal loss under the stability model, a novel high-sensitivity detection technique based on the narrow-band mixing-frequency theory was proposed.

**Results and Discussion:**

*In vitro* experiments demonstrated that only SPIONs with reduced core size and increased hydrodynamic diameter could exhibit relatively stable signals, consistent with the theoretical predictions, under strong interference environments. Further validation via a rabbit mammary sentinel lymph node (SLN) experiment demonstrated that the self-developed SPION carrier (with a core size of 20 nm and a hydrodynamic diameter of 80 nm) achieved long-term stable signal output in living animals, with its sensitivity reaching an acceptable level in the MPI detection system.

## 1 Introduction

MPI is a new *in vivo* quantitative imaging method that uses SPIONs as tracers, and it has been widely applied for clinical applications such as tumor, cardiovascular, and functional metabolism diagnosis due to its high sensitivity and safety features (Gleich and Weizenecker, 2005; Goodwill et al., 2009; Knopp et al., 2010; Goodwill and Conolly, 2011; Othman et al., 2012; Vogel et al., 2023). Superparamagnetic iron oxide nanoparticles (SPIONs) have rapidly emerged recently in the field of medical detection and therapy with the advantages of excellent biocompatibility, high magnetization, and tunable surface functionalization, which have been widely applied in diverse fields, including drug delivery, magnetic particle imaging (MPI), hyperthermia, and immunoassay.

**FIGURE 1:**
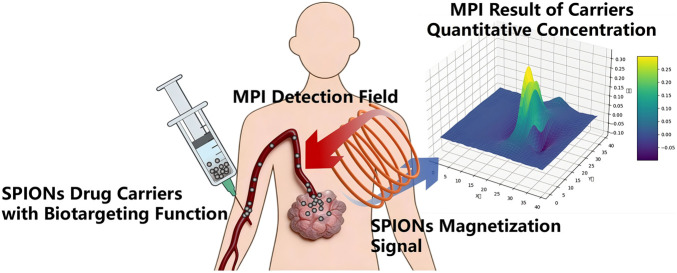
Schematic diagram of drug loading and quantitative characterization of SPION carriers.

SPIONs exhibit remarkable advantages as carriers for drug delivery: their superparamagnetism enables targeted drug delivery under the mediation of an external magnetic field; their surface can be simultaneously modified using multiple functional groups, endowing them with both biotargeting and therapeutic capabilities; their nanoscale characteristics facilitate the crossing of the blood–brain barrier, providing an efficient approach for intracranial drug delivery. In addition, with the support of MPI detection, SPIONs allow visual and quantitative evaluation of the *in vivo* distribution of loaded drugs, thereby guiding the optimization of therapeutic regimens, as shown in Figure 1. Therefore, SPION-based carriers are recognized as promising novel delivery systems with great clinical translation potential.

MPI is a new *in vivo* quantitative imaging method that uses SPIONs as tracers, and it has been widely applied for clinical applications such as tumor, cardiovascular, and functional metabolism diagnosis due to its high sensitivity and safety features (Gleich and Weizenecker, 2005; Goodwill et al., 2009; Knopp et al., 2010; Goodwill and Conolly, 2011; Othman et al., 2012). Basically, the signal of MPI is the nonlinear magnetization response of SPIONs under a rapidly changing AC field (Graeser et al., 2019; Mason et al., 2017). However, based on our latest understanding of superparamagnetism, the dynamic behavior of SPIONs may be affected by a series of physical and biological factors, including the parameters of the AC field, material properties, and *in vivo* environmental disturbances.

Dual frequency is a classical technique for small nonlinear harmonic detection, which offers significant signal-to-noise ratio (SNR) advantages compared to the conventional single-frequency method (Thomas et al., 1976; Warrenw and Hewlett, 1948; Welford, 1985). Although MPI improves the SNR by filtering fundamental noise and harmonic signal acquisition, the excitation system (power supplies and amplifiers) inevitably generates harmonics using nonlinear devices inside, leading to measurement errors. Dual frequency is an effective method for mitigating these intrinsic harmonic interference effects to enhance MPI sensitivity (Goodwill et al., 2009; Tu et al., 2014; Tu et al., 2012; Krause et al., 2007; Nikitin et al., 2007). Previous MPI or magnetic particle spectroscopy (MPS) dual-frequency system designs typically employed a high-frequency field of kHz and a low-frequency field of tens to hundreds Hz (Goodwill et al., 2009; Tu et al., 2014). However, since the third harmonic (*f*_1_+2*f*_2_) closely approaches fundamental frequencies *f*_1_, high-frequency noise levels must remain under control. In this study, we designed new dual-frequency parameters by appropriately increasing the low-frequency components to the kHz levels, along with a narrow-band design, achieving a detection limit of 0.273 ngFe for the third harmonic response under single-side detection systems.

For SPION carriers, stable signal output is essential for the accurate quantification of local concentration. Most studies on nanocarriers focused on surface modification and the optimization of drug delivery efficiency. In terms of concentration detection, previous research focused on AC magnetic susceptibility and relaxation effects (Zhi et al., 2021; Song et al., 2020; Dogan et al., 2022), while studies on accurate concentration reconstruction under complex interference conditions remain unexplored, which may be caused by the complexity and non-quantifiability of *in vivo* interferences; for example, the intensity of surface potential of most biomodified SPIONs would decrease when the pH environment is close to the isoelectric point, resulting in changes in particle dispersion, nonspecific agglomeration, and relaxation characteristics. Notably, although environmental factors directly affect the outer layer of SPIONs, they still inevitably lead to undulation of dynamic magnetization behavior (Menghao et al., 2024; Jiménez-Villacorta et al., 2010). Due to the complex environment of human tissues, blood, cells, proteins, and their changes caused by diseases, these kinds of *in vivo* interferences are almost unavoidable in SPION carrier detection, which lead to errors in the evaluation of carrier concentration (Hamed et al., 2015).

We here proposed a key hypothesis based on the third harmonic alternating current susceptibility (ACS) model of SPION carriers: when SPION carriers with specific sizes are matched to the frequency of the MPI detection system, a relatively stable state emerges, enabling accurate quantitation of the carriers. To validate this hypothesis, the research team conducted independent analyses on nanoparticles of different sizes across various frequency bands under a series of interference factors. Meanwhile, the analysis results were verified in whole-blood samples, ultimately leading to the development of a mathematical model. This model can quantitatively evaluate the fluctuation range and signal loss of SPION carriers under MPI characterization once the particle-device parameters are determined. The magnetization model and *in vitro* experimental results demonstrate that 20-nm particles can provide a stable signal, whereas signal loss is also induced concomitantly, leading to a loss in system sensitivity. Therefore, we further designed a frequency-mixing narrow-band MPI signal acquisition system and verified the stability of *in vivo* signals of SPION carriers, along with the reliability of the MPI detection system in experiments of sentinel lymph node (SLN) detection in rabbits.

## 2 Analysis of bio-physical influencing factors in *in vivo* SPIONs

### 2.1 Basic structure and *in vitro* ACS stability

Biofunctionalized SPIONs are mainly composed of three layers, namely, the superparamagnetic core with a diameter *d*_c_, the hydrophilic coating with a diameter *d*_h_, and surface modifiers. The magnetic core provides the basic superparamagnetic response, the hydrophilic coating provides surface physical and biological properties such as the functional –COOH group and –NH_2_ group, and surface modifiers provide rich biological functions such as targeted molecules and drugs.

For the *in vitro* environment, whether in pure water or low-concentration buffers, SPIONs can generally maintain their dispersed state due to the electrostatic repulsion or space occupancy between neighboring particles. Consequently, the ACS and its harmonic components can maintain stable values (Yoshida et al., 2019; Gutiérrez et al., 2011), at which the MPI detection system can accurately evaluate the concentration of SPION carriers via their AC magnetization signals. However, for *in vivo* applications, SPIONs may be affected by multiple complex factors simultaneously, leading to unquantifiable changes in the ACS of SPION carriers, which brings challenges to the quantitation of the carrier concentration (Gutiérrez et al., 2011).

### 2.2 Basic structure and *in vitro* ACS stability

**FIGURE 2:**
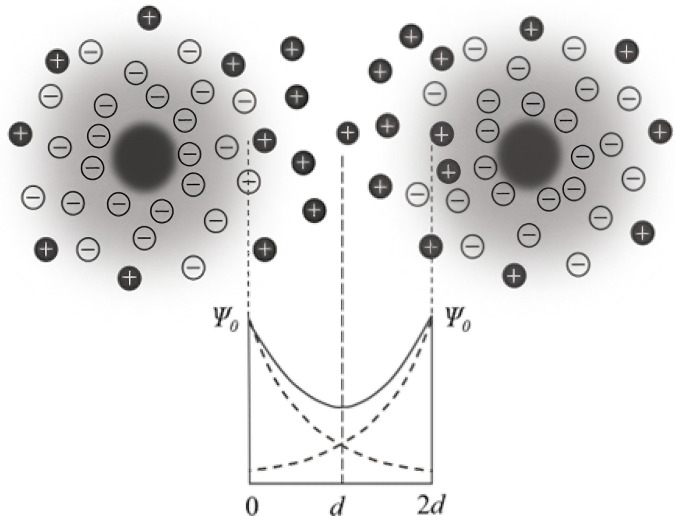
Distribution of the electric double layer of two particles in a steady state in suspension and the surface potential *Ψ*_0_ as a function of distance. The dotted line shows the attenuation of the surface potential with distance for a single particle.

An important way in which SPIONs lose their stability is through ion and pH interference in blood and tissues. When the outer layers of SPIONs come into contact with an aqueous electrolyte, dissociation reactions can generate an electric surface charge (Delgado et al., 2007; Zukoski and Saville, 1986). Ions in the liquid reorganize to form a nanometric layer that balances the surface charge, known as the electrical double layer (EDL) (Remco et al., 2018), as shown in Figure 2. The electrostatic repulsion *V*_r_ must always be larger than the van der Waals force *V*_a_, i.e., *V*_r_ > *V*_a_. The value of *V*_r_ per unit area at room temperature (25 °C) is given by the following equation.

$$V_{r}=\frac{4.82\times 10^{-3}\sqrt{c}}{v}\gamma^{2}\exp\left(-2\kappa d\right),$$ (1)

$$\gamma =\frac{\exp\left(z/2\right)-1}{\exp\left(z/2\right)+1},Z=\frac{ve\psi_{0}}{kT},$$ (2)

where *v* is the atomic valence number, 2*d* is the distance between electrical bilayers of neighbor particles, and *κ* is the Debye constant, which can be determined by the number of counterions per unit volume, atomic valence, and the dielectric constant of the solution. The surface potential can be calculated as follows: *Ψ*_0_ = 0.059 (pH_0_ − pH), where pH_0_ is the pH of the solution when *Ψ*_0_ is 0. Here, *Ψ*_0_ is determined by both the electric charge of SPION materials and the ionic concentration *c* of the environment solution, which contribute the anti-ion cloud and bilayers to maintain the distance between neighboring particles.

The van der Waals force *V*_a_ between two particles per unit area is given by

$$V_{a}=-\frac{A}{48\pi d^{2}},$$ (3)

where A is Hamaker’s constant, which determines the van der Waals force between macroscopic objects, varying from substance but with a value of approximately 10^−20^ J, and 2*d* is the distance between electrical bilayers of neighbor particles. Either the solution ion or environment pH could affect electrostatic repulsion and particle stability, both of which are always uncertain in the human body. For instance, myoblast usually shows a large number of Ca^2+^ on their surface, and the tumor area always shows a lower pH than the normal tissue area. Therefore, an ACS experiment was conducted under different ion concentrations to evaluate the stability of SPIONs under different pH environments; both factors show similar effects on ACS due to the isoelectric point effect. Moreover, greater surface potential is also expected for SPIONs manufacturing, which is relative with the electric charge of core and surface materials.

### 2.3 Protein and cell interference

In addition to the ion concentration interference, SPIONs could also be immobilized by other biological effects, such as cell adsorption and protein unspecific binding. Due to the small size of superparamagnetic SPIONs, they can be adsorbed through several biological mechanisms, among which adsorptive-mediated transcytosis (AMT) is the most common method of adsorption (Pinheiro et al., 2021; Cláudia et al., 2016). Nonspecific binding always occurs much faster than cell adsorption, usually less than 30 min.

Menghao et al. (2024) from Yokohama National University found that the ACS of immobilized SPIONs strongly depends on the orientation of the easy magnetization axis; for SPIONs with randomly arranged easy magnetization axes, the magnetic susceptibility can be expressed as follows (Tu et al., 2014):

$$\chi \left(\omega \right)=\frac{1}{3}\left(\frac{\chi_{∥}}{1+i\omega \tau_{∥}}{+2\chi }_{⊥}\right),$$ (4)

where the longitudinal component *χ*_∥_ corresponds to the reversal of magnetization due to thermal fluctuations and the transverse component *χ*_⊥_ corresponds to the alignment of moment along the internal effective field with precession. The immobilized SPIONs lead to a change in the characteristics of its magnetized axis and the responsiveness of the ACS.

In this study, different concentrations of glycerol were used to simulate SPION immobilization, while different concentrations of serum and whole blood were used for cell adsorption and nonspecific binding interference.

### 2.4 Magnetic relaxation

For independent nanoparticles, the Néel and Brownian relaxation effects can be defined as follows (Ota and Takemura, 2019):

$$\tau_{B}=\frac{3\eta_{0}V_{h}}{k_{B}T},$$ (5)

$$\tau_{N}=\frac{1}{f_{0}}\exp\left(\frac{K_{a}V_{c}}{k_{B}T}\right),$$ (6)

where *τ*_B_ is the Brownian relaxation time, *τ*_N_ is the Néel relaxation time, *η*_0_ is the viscosity of the carrier fluid, *V*_h_ is the hydrodynamic volume of the particle with the hydraulic diameter *d*_h_, *V*_c_ is the volume of the superparamagnetic core with the core diameter *d*_c_, *K*_a_ is the anisotropy constant, *k*_B_ is the Boltzmann constant, *T* is the absolute temperature, and *f*_0_ ≈ 10^9^ Hz is a typical attempt time. The SPION response can only be detected under the condition of *τ*_effm_ = *τ*_N_ × *τ*_B_/(*τ*_N_ + *τ*_B_) << 1/2π*f*_exc_, where *f*_exc_ is the frequency of the excitation field.

It must be noted that only when *τ*_eff_ is much smaller than 1/2π*f*_exc_, the AC magnetization response can maintain its stability for some frequency variation in the nearby interval (equal to diameter variation), as shown in Figure 2, which is one of the important factors affecting the quantitative accuracy of MPI.

### 2.5 Phase delay and complex magnetization

The relaxation effect near 2π*f*_exc ×_ *τ*_eff_ = 1 separates the ACS response into two parts, i.e., the real (in-phase) part *χ′*(*ω*), which provides responses in real time with the AC magnetic field, and the imaginary (out of the phase) part *χ″*(*ω*), which shows a phase lag with the AC magnetic field. The real and imaginary ACS can be simply expressed using the Debye theory (Tong et al., 2004):

$$\frac{\chi^{\prime }\left(\omega \right)}{\chi_{0}}=\frac{1}{\left(1+{\left(\omega \tau_{\text{eff}}\right)}^{2}\right)},$$ (7)

$$\frac{\chi^{\prime \prime }\left(\omega \right)}{\chi_{0}}=\frac{\omega \tau_{\text{eff}}}{\left(1+{\left(\omega \tau_{\text{eff}}\right)}^{2}\right)},$$ (8)

$$\chi \left(\omega \right)=\left|\chi^{\prime }\left(\omega \right)+i\chi^{\prime \prime }\left(\omega \right)\right|=\sqrt{{\left(\chi^{\prime }\left(\omega \right)\right)}^{2}+{\left(\chi^{\prime \prime }\left(\omega \right)\right)}^{2},}$$ (9)

where *χ*_0_ is the DC static susceptibility and *ω* is the angular frequency of the AC magnetic field. Moreover, in MPI, we used harmonic signals, instead of fundamental response, for noise suppression. Especially, in this study, we used the narrow-band third harmonic signal to enhance the SNR. The third harmonic component of *χ′*(*ω*) and *χ″*(*ω*) can be calculated using the following empirical equations (Yoshida and Enpuku, 2009):

$$\left|\chi_{3}\right|=\frac{\chi_{3}\left(0\right)}{1+{\left[k_{m3}\left(\xi_{a}\right)\omega \tau_{eff}\right]}^{2}},$$ (10)

$$\theta_{3}=3\tan^{‐1}\left[k_{\theta 3}\left(\xi_{a}\right)\omega \tau_{eff}\right],$$ (11)

with Equations 12–14:

$$\frac{\chi_{3}\left(0\right)}{\chi_{0}}=\frac{3\xi_{a}^{2}}{180+12.2\xi_{a}+13.2\xi_{a}^{2}+2.29\xi_{a}^{3}},$$ (12)

$$k_{m3}\left(\xi_{a}\right)=2.15‐\frac{0.145\xi_{a}^{2}}{1+0.187\xi_{a}+0.0683\xi_{a}^{2}},$$ (13)

$$k_{\theta 3}\left(\xi_{a}\right)=1.31‐\frac{0.0496\xi_{a}^{2}}{1+0.222\xi_{a}+0.0435\xi_{a}^{2}}.$$ (14)

**FIGURE 3:**
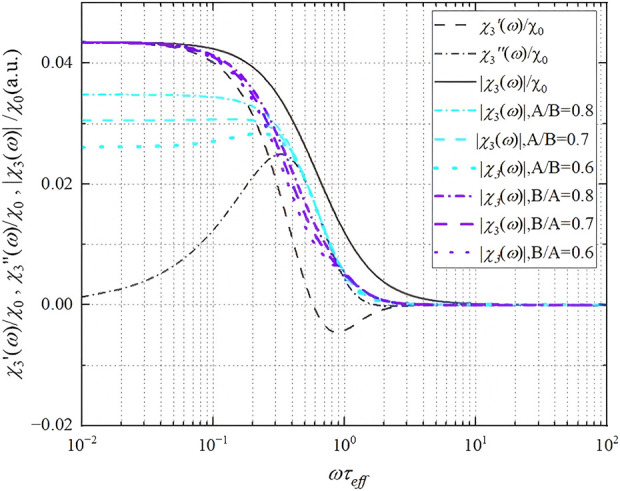
The solid, dashed, and dashed and dotted lines in gray color represent the absolute third harmonic susceptibility |*χ*_3_(*ω*)|, real third harmonic susceptibility *χ*_3_ *'*(*ω*), and imaginary third harmonic susceptibility *χ*_3_ *”*(*ω*) calculated using the Debye model, respectively, under the condition of *ξ*_a_ = 2. The blue and purple lines show the absolute third harmonic susceptibility |*χ*_3_(*ω*)| values with different *χ*_3_ *’*(*ω*)_peak_/*χ*_3_ *”*(*ω*) _peak_ ratios; A presents the dimensionless coefficient of *χ*_3_ *’*(*ω*)_peak_, and B presents the dimensionless coefficient of *χ*_3_ *”*(*ω*) _peak_.

Here, *χ*_3_(0) is the susceptibility in the case of frequency approach to 0, *ξ*_a_ is a variable parameter from the Langevin equation, *M*(*t*) = *mL*(*ξ*_a_2*πft*), which is related to the magnetic moment *m* of particles and the temperature of the environment, and *k*_*m*3_(*ξ*_a_) and *k*_*θ*3_(*ξ*_a_) are hypothetical parameters related to *ξ*_a_; the expressions of *k*_*m*3_(*ξ*_a_) and *k*_*θ*3_(*ξ*_a_) with respect to *ξ*_a_ are obtained through numerical simulation, as given by Equations 13, 14. The gray line in Figure 3 shows the *χ*_3_′(*ω*) and *χ*_3_″(*ω*), and |*χ*_3_(*ω*)| = (*χ*_3_′(*ω*)^2^ + *χ*_3_″(*ω*)^2^)^1/2^, where *ξ*_a_ = 2.

As shown in Figure 3, |*χ*_3_(*ω*)| shows a monotonically decreasing trend from 2π*f*_exc ×_ *τ*_eff_ = 10^−1^ to 10^1^, while *χ*_3_′(*ω*) and *χ*_3_″(*ω*) show the decreasing and increasing trends, respectively. This result implies that, regardless of *χ*_3_′(*ω*), *χ*_3_″(*ω*), or |*χ*_3_(*ω*)|, signal stability cannot be achieved within the interval from 2π*f*_exc ×_ *τ*_eff_ = 10^−1^ to 10^1^. Considering the relationship between *d*_h_^3^ and *τ*_B_, along with *e*(*K*_a_ *d*_c_^3^) and *τ*_N_, the frequency change in 2π*f*_exc ×_ *τ*_eff_ = 10^−1^ to 10^1^ corresponds to an approximately 2–3 times change in the equivalent particle diameter. Notably, in most MPIs, we tend to use a high-frequency AC field between 2π*f*_exc ×_ *τ*_eff_ = 10^−1^ to 1 and particle size with a large saturation magnetization to generate a higher absolute intensity of the induced signal. For instance, the Néel relaxation time of an independent 25-nm Fe_3_O_4_ core SPIONs with an anisotropy energy density *K* = 13 kJ/m^3^ is approximately 0.5 × 10^−5^ s, corresponding to a frequency *f* of approximately 31.8 kHz, which is close to the safe frequency of MPI from 20 to 25 kHz, suggesting that the signal intensity is highly sensitive to the relaxation changes around these frequencies.

An interesting phenomenon is that the peak values of *χ*_3_ *′*(*ω*) and *χ*_3_″(*ω*) are affected by several properties of SPIONs and the external excitation field. For instance, a higher *µ*_*0*_ *mH*/*k*_b_ *T* tends to increase the rates of *χ*_3_′(*ω*)_peak_ and *χ*_3_″(*ω*)_peak_, resulting in some changes in |*χ*_3_ (*ω*)| to a different stability. However, besides the external field and *m*, the relaxation changes and phase delay seem to be the dominant influencing factors, which are always changing in the human body. As shown in Figure 3, |*χ*_3_(*ω*)| is also calculated with different *χ*_3_′(*ω*)_peak_/*χ*_3_″(*ω*)_peak_ ratios. Here, A presents the dimensionless coefficient of *χ*_3_′(*ω*)_peak_, and B presents the dimensionless coefficient of *χ*_3_″(*ω*)_peak_, both of which are always changing due to many parameters of nanomaterials and excitation fields, such as single-domain magnetic moment and field strength. We found that, under some certain conditions, |*χ*_3_(*ω*)| no longer shows monotonically increasing or decreasing trends but remains at a relative stable state within the range of 2π*f*_exc ×_ *τ*_eff_ = 10^−1^ to 10^1^. We consider this the key to improving the stability of the MPI signal, as well as keeping the balance between signal strength and signal stability, based on which we proposed the later MPI stability models.

### 2.6 Magnetic dipole–dipole interaction

For *in vivo* applications, the thickness of the SPION coating needs to be strictly designed to ensure the biological effects associated with an inevitable magnetic dipole–dipole (MDD) interaction. This kind of effect is often ignored in some *in vitro* environment studies, where SPIONs maintain a good dispersion state; however, the agglomeration of SPIONs in the *in vivo* environment was inevitable (Neeharika et al., 2021).

The complexity of the MDD interaction is that for small core size SPIONs, this kind of interaction may enhance the magnetic moment coordination, that is, the equivalent single-domain magnetic moment is increased; at the same time, the increased relaxation time does not exceed the range of serious interference to the signal so that the MPI signal is optimized, and multi-core particles and chain-like particles optimized MPI signals following this principle (Jiménez-Villacorta et al., 2010). However, for some larger core size SPIONs or for some small particles that undergo severe agglomeration, the increase in equivalent particle size may directly disrupt the coordination of the relaxation time with the AC field, which makes it impossible for SPIONs to achieve fast spin under the AC field, leading to a significant decrease in the MPI signal (Kolja, 2017; Kai et al., 2019).

A thicker coating layer can obviously help prevent the occurrence of an unstable MDD interaction. However, in many *in vivo* applications, it is necessary to maintain a certain hydrodynamic diameter to maintain the biological effects. For example, only SPIONs with a hydrodynamic diameter smaller than 150 nm can penetrate the basement membrane of the lymph vessel. Similarly, SPIONs with a hydrodynamic diameter between 10 nm and 100 nm can easily penetrate the blood–brain barrier (Hersh et al., 2022; Cláudia et al., 2016). In addition, the total amount of coating materials, such as dextran, is always strictly regulated in clinical practice, suggesting that if the hydrodynamic diameter is larger, the relative Fe content must be lower, which also causes less signal strength.

We believe that a reasonable solution is to moderately increase the hydrodynamic diameter while ensuring the biological effects and provide an appropriate signal strength compensation method for the reduction in the Fe content.

### 2.7 Theoretical model of expected SPIONs

Based on the calculation and analysis mentioned above, we proposed a theoretical model for quantitative analysis of SPIONs, which should first satisfy the basic requirements:

$$d_{\text{dipole}}<d_{H}<d_{\text{target}}\text{ and }d_{\text{mesurement}\;}<d_{c}<d_{\text{relaxtion}},$$ (15)

where *d*_H_ represents the hydrodynamic diameter, *d*_dipole_ represents the minimum hydrodynamic diameter that prevents or partially prevents the occurrence of the MDD interaction affecting the ACS magnetization response, and *d*_target_ is the maximum hydrodynamic diameter at which SPIONs do not lose their biological functions. *d*_c_ represents the core size, *d*_mesurement_ is the smallest core diameter corresponding to its lowest single-domain magnetic moment that satisfies the sensitivity requirement of the MPI system, and *d*_relaxtion_ is the maximum core diameter that satisfies the basic relaxation and frequency conditions, both of which should be discussed within the safe and achievable range of the MPI system, generally not exceeding 20 kHz and a 6 mT_rms_ excitation field (Emine et al., 2012; Reilly, 1989; Reilly, 1998).

Based on the analysis above, a theoretical model was developed to describe the stability and necessary signal loss of the MPI proposed, which provided a compromised way to develop suitable and quantifiable SPIONs and the MPI system:

$$\sigma =\frac{\left|\left(\chi^{\prime }\left(\omega \right)-i\chi^{\prime \prime }\left(\omega \right)\right)\right|\omega ={2\pi fx}_{1}-\left|\left(\chi^{\prime }\left(\omega \right)-i\chi^{\prime \prime }\left(\omega \right)\right)\right|\omega ={2\pi fx}_{2}}{\left|\left(\chi^{\prime }\left(\omega \right)-i\chi^{\prime \prime }\left(\omega \right)\right)\right|\omega ={2\pi fx}_{1}}\times 100\%,$$ (16)

$$\frac{V_{fxl}}{V_{{fxl}_{0}}}=\frac{\omega_{fxl}}{\omega_{{fxl}_{0}}}\cdot \frac{Q_{fxl}}{Q_{{fxl}_{0}}}\cdot \frac{\left|\chi_{fxl}\right|}{\left|\chi_{fxl_{0}}\right|},$$ (17)

$$\frac{M}{M_{0}}\frac{mL\left(\xi \right)}{M_{0}L\left(\xi_{0}\right)}\propto \frac{n}{n_{0}}\propto \frac{d_{C}^{3}M_{S}L\left(\xi \right)}{d_{C_{0}}^{3}M_{S_{0}}L\left(\xi_{0}\right)}\cdot \frac{n}{n_{0}}\approx \frac{M_{S}L\left(\xi \right)}{M_{S_{0}}L\left(\xi_{0}\right)},$$ (18)

$$\frac{M_{dH}}{M_{dH_{0}}}\propto \frac{\varrho \left(Fe\right)}{\varrho_{0}\left(Fe\right)}\propto \frac{d_{c}^{3}\left(d_{H}^{3}‐d_{c}^{3}\right)}{d_{c_{0}}^{3}\left(d_{H_{0}}^{3}‐d_{c_{0}}^{3}\right)}.$$ (19)

Equation 16 presents a basic model that quantified the fluctuations of absolute susceptibility according to Figure 3. Here, *σ* is the target fluctuating coefficient, which represents the signal fluctuation caused by magnetic susceptibility changes under various external interferences, *f*_*x*1_ and *f*_*x*2_ are two arbitrary points in the frequency interval of [*f*_*xl*,_ *f*_*xh*_], where *f*_*xl*_ is the standard frequency that the MPI expects to use satisfying *f*_*xl*_ ≤ 1/π*τ*_eff_ and ≤20 kHz when SPIONs are extremely dispersed, and *f*_*xh*_ is the frequency when SPIONs start aggregating to a limited state from the initial dispersed state. In the case of 2- or 3-fold changes in equivalent particle size, considering relaxation, magnetic moment, and MDD interactions, it is necessary in practice to set *f*_*xh*_ ≈ 10 × *f*_*xl*_ or higher. Notably, either *f*_*xl*_ or *f*_*xh*_ should be discussed under different excitation strengths ≤ 3 mT.

As for the utilization of the inductive MPI sensor, i.e., the pick-up coil, we use a high frequency to improve system sensitivity. Therefore, a large *f*_*xl*_ close to 20 kHz was used in this research, which needs optimized SPIONs parameters considering both the biological and physical factors. However, in any case, a certain compromise in signal sensitivity may still be necessary, including compromises of AC frequency, core size of SPIONs *d*_*c*_, or hydrodynamic size *d*_H_.

Equation 17 presents an absolute signal loss when a lower *f*_*xl*_ is selected to ensure the stability of *σ* in Equation 16. Here, the signal loss is calculated for harmonic MPI case (*Q* = 1) and narrow-band MPI (*Q* = *ωL*/*R*). The signal voltage detected by the pick-up coil decreases with *f*_*xl*_ for harmonic MPI, while the signal voltage decreases with *f*_*xl*_^*2*^ for narrow-band MPI because a resonant circuit with a quality factor *Q* is always used to enhance the designated frequency.

Equations 18, 19 present the equivalent signal loss due to the compromise in frequency, *d*_*c*_ and *d*_H_, respectively. Equation 18 presents the loss of SPION magnetization due to the reduction in the single-domain magnetic moment if a small *d*_*c*_ is used to ensure the stability of *σ*. It is important to note that the FDA approval always specifies the maximum Fe dose that can be injected. Therefore, the total magnetization does not decrease with *d*_c_^3^, but in the form of saturation magnetization and Langevin function.

Equation 19 presents the loss of SPION magnetization caused by a thicker hydrophilic coating to avoid MDD interference. Although the hydrophilic coating does not directly provide the strength of the magnetization response, clinical approval always specifies the maximum injection mass of coating, such as dextran or PEG. Therefore, if the coating is thick, the relative concentration of Fe decreases, which influences the signal intensity.

As a short summary, SPIONs should simultaneously meet the basic physical and biological applicable conditions given in Equation 15 and conduct the signal fluctuation research, testing, and evaluation based on Equation 16. Then, adjustments to *f*_*xl*_, *d*c, *d*_h_, and their interference on the signal strength should be made according to Equations 17–19, under different excitation strengths less than 6 mT_rms_.

## 3 Stability characterization experiments of clinical-grade magnetic nanomaterials

### 3.1 SPION samples

**FIGURE 4:**
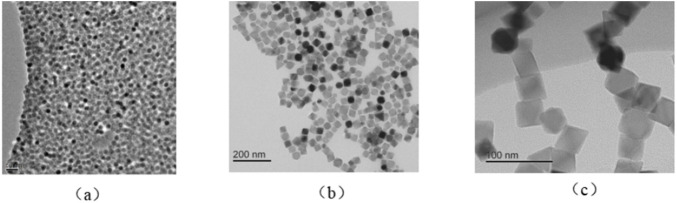
**(A)** TMS result of tracer 1; **(B)** TMS result of tracer 2; **(C)** TMS result of tracer 3.

Four types of SPION samples were used in stability characterization experiments. Tracers 1–3 were self-developed based on Equations 16–19, with different core diameters, hydrodynamic diameters, and surface potentials, and were prepared using the co-precipitation method. Three tracers were produced in a GMP-grade workshop (Nanoeast Technology, China) to ensure *in vivo* safety. Tracer 4 was Resovist (MRI tracer, Fuji film, Japan), which has been considered a representative stable tracer until now due to its multi-core structure. Detailed parameters of four SPIONs samples are listed in Table 1. The TMS characterization results of the three tracers co-developed with Nanoeast are shown in Figure 4.

**TABLE 1:** Parameters of the four tracers.

Name	Tracer 1	Tracer 2	Tracer 3	Tracer 4
Producer	Self-developed	Self-developed	Self-developed	Resovist
Inner diameter	20 nm (single-core)	25 nm (single-core)	30 nm (single-core)	21.6 nm (multi-core)
Outer diameter	60 nm	110 nm	200 nm	68 nm
Magnetization	Medium	Large	Large	Large
$\tau_{B}$	Medium	Large	Large	Medium
$\tau_{N}$	Medium	Large	Large	Medium
Nuclear interaction	Small	Medium	Medium	Small
Surface modification	Dextran	Dextran	Dextran	Dextran
COOHization	√	√	√	√
Zeta potential	−35.2 mV	−51.7 mV	−55.4 mV	−30.7 mV

### 3.2 Magnetic particle spectroscopy and hydrodynamic diameter characterization methods

The nonlinear AC response of SPIONs was characterized using a home-made magnetic particle spectroscopy (Magnetic Particle Spectroscopy) device, which had been approved by the Chinese NMPA for immunoassay usage (JY-01, Jiayin Technology, China). The MPS device can provide an AC excitation field with strengths ranging from 0 mT_rms_ to 6 mT_rms_ and frequency ranging from 0 kHz to 20 kHz. The third harmonic signal was detected using a low-noise narrow-band detection system, with a maximum SNR of more than 40,000 for 400 *µ*g Resovist. In this work, the excitation field strength was fixed to 2.8 mT_rms_, while the frequency was used from 1 kHz to 20 kHz. The third harmonic effective value |*V*_3_| was detected as the main magnetization signals. The Fe content of each sample was fixed to 400 *µ*g, except for special declaration. The hydrodynamic diameter and the surface potential were tested using a Dynamic Light Scattering Tester (Malvern Paralytical, Nano ZS + MPT-2).

### 3.3 Interference methods

Ion interference: SPIONs diluted with 100 µL pure water were mixed with 100 *µ*L 2× PBS, 20× PBS, and 200× PBS to achieve a final PBS concentration of 1×, 10×, and 100×, respectively. The different Na^+^ ions in PBS resolution can verify the interference of Na^+^ ions on the stability of SPIONs.

Viscosity interference: SPIONs diluted with 100 *µ*L pure water were mixed with 5 *µ*L glycerol to achieve a solution viscosity of 1.18 mPa·s so as to imitate the effect of blood viscosity.

Serum composite interferences: SPIONs diluted with 100 *µ*L pure water were mixed with 100*µ*L horse serum (BL209A, Biosharp, China) to achieve a 50% concentration serum interference. Rich ions, proteins, and exosomes may affect the stability of SPIONs simultaneously.

Whole-blood composite interference: SPIONs diluted with 100 *µ*L pure water were mixed with 10 μL, 50 μL, 100 μL, and 300 *µ*L of rabbit whole blood to imitate different interference levels of whole blood. Ion, proteins, cells in the whole blood, and blood viscosity might affect the stability of SPIONs simultaneously.

To rule out the possibility of more interfering factors, such as temperature changes and blood clotting, all experiments were set in a 295 K environment, and all interfering measurements were obtained 30 min after mixing.

### 3.4 Frequency dependence of *V*_3_rms on Na^+^ relative to pure water

**FIGURE 5:**
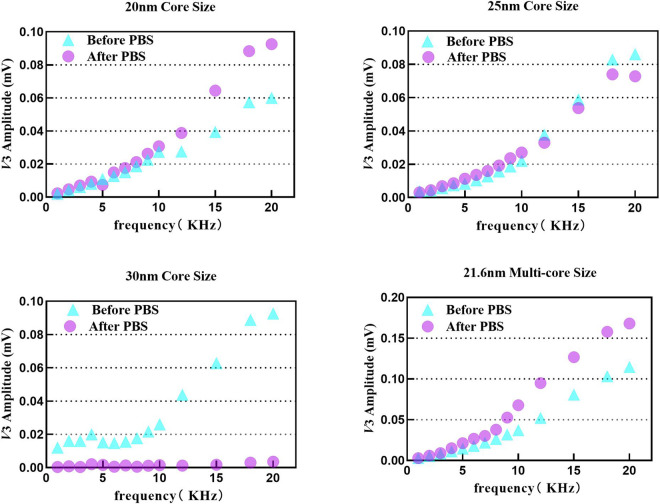
*V*_3rms_ variation in tracers 1–4 under pure water and 1× PBS from 1 kHz to 20 kHz.

Figure 5 shows the frequency dependence of *V*_3_rms, i.e., the signal at frequency *f*_1_+2*f*_2_ of four tracers in pure water and physiological Na^+^ ion concentration. The initial SPION suspension was prepared in pure water. The Fe content of the samples was 400 μg, the excitation intensity was 2.8 mT_rms_, the excitation frequency was set from 1 kHz to 20 kHz, and the test temperature was 295 K.

Tracers 1, 2, and 4 showed good stability in 1× PBS buffer, especially tracer 2, which remained almost unchanged after the interaction. Tracer 3 almost lost its magnetization response in all frequency bands after 1× PBS interference, indicating that the 30 nm magnetic core diameter is too large for *in vivo* MPI imaging. Interestingly, both tracer 1 and tracer 4 showed increased signals after 1× PBS interference. We propose two reasons. First, the pure water solution might not be able to effectively provide enough negative ions to completely form the electrical double layer, but the low-concentration PBS does. Second, the fluctuation of *α* given in Equation 15 is not monotonically decreasing, causing a slight increase in the signal.

### 3.5 Frequency dependence of *V*_3_rms on viscosity

**FIGURE 6:**
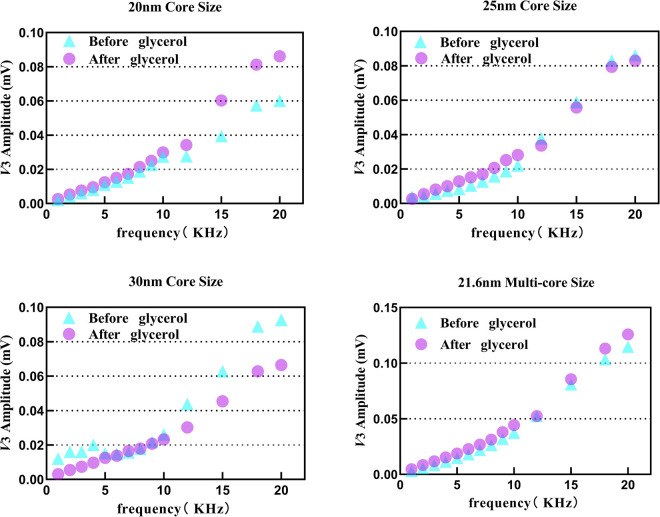
*V*_3rms_ variation in tracers 1–4 after adding 5 *µ*L glycerol from 1 kHz to 20 kHz.

We next measured the *V*_3_rms of four tracers in pure water and a mixture of water and 5 µL glycerol; the viscosity of the glycerol–water mixture was 1.02 mPa·s (measured at 295 K), as shown in Figure 6. The initial SPION suspension was prepared in pure water. We found that all *V*_3rms_ signals of four tracers maintained a relatively stable trend especially for tracer 2 and tracer 4. In more detail, tracer 3 showed a slight decrease in the signal under viscosity interference, which increased with the excitation frequency, reaching approximately 35% at 20 kHz. Moreover, similar to ionic interference, the *V*_3rms_ of tracer 1 increased slightly. However, we can consider that physiological blood viscosity does not lead to the occurrence of severe signal fluctuations at approximately 10 kHz.

### 3.6 Frequency dependence on serum

**FIGURE 7:**
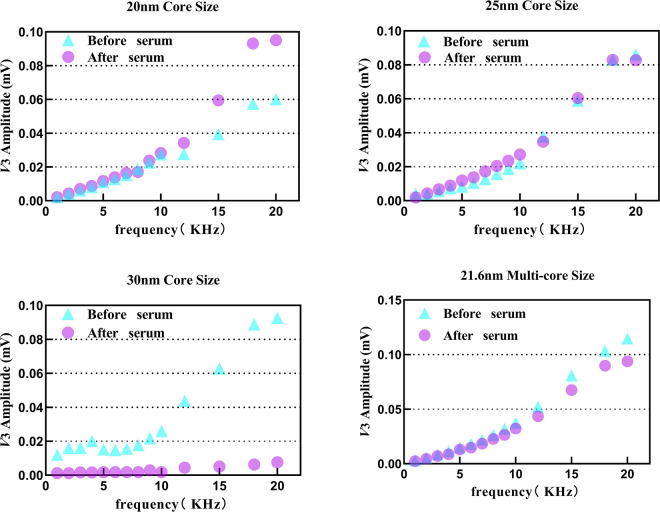
*V*_3rms_ variation in tracers 1–4 after adding 100 *µ*L horse serum from 1 kHz to 20 kHz.

Although ion concentration and viscosity showed their own interference effects, a more complex interference is still needed to simulate the actual *in vivo* environment. Figure 7 shows the variation in *V*_3rms_ of four tracers before and after adding 100 *µ*L serum. The initial SPION suspension was prepared in pure water.

Tracers 1–3 showed the same signal characteristics as it in 1× PBS interference, but the *V*_3rms_ of tracer 4 decreased slightly after serum mixture, suggesting that the influence of serum is significantly stronger than that of ions alone. Horse serum not only confirmed protein interference but also revealed a certain amount of salt ions, necessitating comprehensive analysis of both factors.

### 3.7 Frequency dependence on whole blood

**FIGURE 8:**
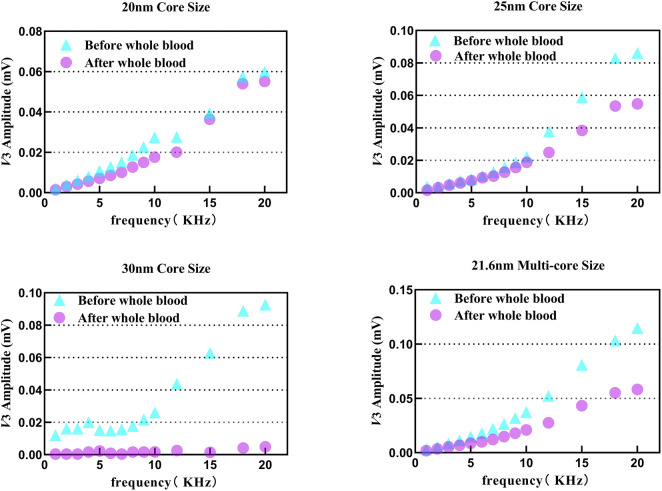
*V*_3rms_ variation in tracers 1–4 after adding 100 *µ*L whole blood from 1 kHz to 20 kHz.

Whole blood contains all the components of serum, along with a large number of blood cells, and its viscosity, etc., is also different from that of serum. Cells may solidify SPIONs through adsorption, phagocytosis, and other processes, which cause changes in their magnetization response behavior. Whole blood can more realistically represent disturbances under the *in vivo* environment than serum. Figure 8 shows the variation in *V*_3rms_ of four tracers before and after adding 100 *µ*L whole blood. The initial SPION suspension was prepared in pure water.

Different from the result under ion and serum interference, all tracers, except for tracer 1, showed signal loss due to the influence of whole blood even if the blood concentration is only 50% in this case. It should be noted that the actual situation of SPIONs in the human body might be even more serious. Fortunately, tracer 1 showed good stability in this case, indicating that the small compromise in terms of magnetic core diameter and magnetic moment, as well as the increase in the coating thickness according to (16–19), has a significant effect.

Table 2 presents the changes in the outer diameter and zeta potential of SPIONs under dynamic light scattering (DLS) characterization before and after 1× PBS and glycerol interference. Since whole blood contains cells, proteins, exosomes, and other components, all of which can cause serious interference with particle size and potential results, and serum contains some protein components that can also cause errors in particle size and potential characterization, we did not analyze particle size and potential changes under serum and whole-blood conditions here. Under PBS interference, both tracer 1 and tracer 4 exhibited a minimal increase in particle size, showing similar phenomena in the third harmonic signal: all demonstrated slight signal enhancement, and their zeta potential also decreased slightly in both two tracers; tracer 2 showed an approximately two-fold increase, while the outer diameter of tracer 3 showed significant growth under 1× PBS interference, which caused signal attenuation to near 0; the zeta potential of tracer 2 and tracer 3 significantly reduced accordingly. Under glycerol interference, four tracers showed an increase in the outer diameter; tracer 1 and tracer 3 exhibited signal enhancement, tracer 2 maintained nearly stable signals, while tracer 4 showed a slight decrease in the signal. The zeta potential also showed corresponding results: potentials of tracer 1 and tracer 4 decreased slightly, while potentials of tracer 2 and tracer 3 decreased significantly. Overall, potential changes directly affected the particle size distribution, thereby influencing signal fluctuations. The smaller-sized tracer 2 demonstrated comprehensive advantages under various interference conditions, while the multi-core particles formed by the aggregation of smaller-sized particles (tracer 3) ranked second to tracer 2.

**TABLE 2:** Variation in the hydrodynamic diameter and zeta potential of four tracers before and after PBS and glycerol interferences.

Characteristic parameters	Peak particle size (nm)			Polydispersity index (PDI)			Zeta potential (mV)		
Particle	Before	After 1× PBS	After glycerol	Before	After 1× PBS	After glycerol	Before	After 1× PBS	After glycerol
Tracer 1	60.65	67.54	84.17	0.255	0.258	0.186	−35.2	−23.1	−27.6
Tracer 2	110.1	254.5	155.6	0.202	0.515	0.176	−51.7	−20.3	−36.1
Tracer 3	195.3	559.4	215.7	0.356	0.284	0.27	−55.4	−23.5	−43.3
Tracer 4	68.48	69.13	83.32	0.207	0.231	0.198	−30.7	−10.5	−21.7

### 3.8 Strong interference experiments

We used a fixed number of interfering substances, which are much weaker than those in the human body. For example, 1× PBS yielded the physiological concentration of Na^+^ ions, but high-valency ions such as Ca^2+^ and Mg^2+^ were ignored, which may destroy the electrical double layer more powerfully. Therefore, we measured some limit conditions using a higher concentration of buffer solution and whole blood to verify the stability of SPIONs in the limited *in vivo* environment.

**FIGURE 9:**
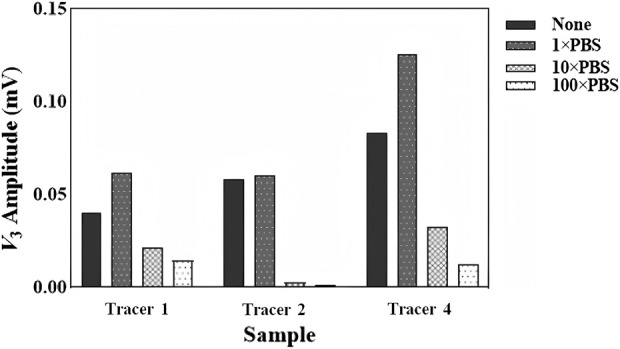
Variation in *V*_3rms_ values of four tracers at 15 KHz and a 2.8 mT AC field under different ion concentrations.

**FIGURE 10:**
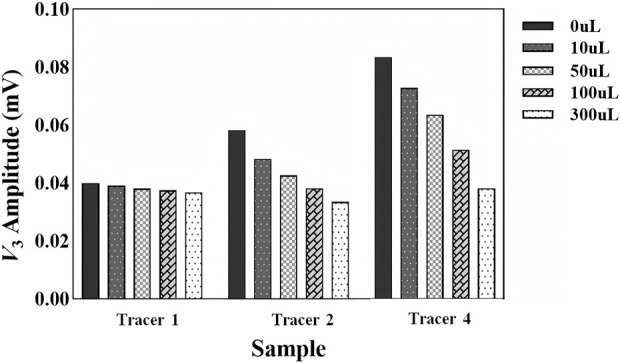
Variation in *V*_3rms_ values of four tracers at 15 KHz and a 2.8 mT AC field after adding different volumes of whole blood.

Figure 9 shows the experiment results in the cases of 1× PBS, 10× PBS, and 100× PBS influences. The amount of the SPION content was fixed at 400 *µ*g and 200 *µ*L, while the excitation frequency was set at 15 kHz. Tracer 3 was excluded from the experiment due to its extreme instability. Figure 10 shows the results of three tracers in the cases of 10*µ*L, 50*µ*L, 100*µ*L, and 300 *µ*L whole-blood interferences. Other experiment conditions were the same as those of the ion interference experiment. Although tracer 2 and tracer 4 showed some instability at high blood concentrations, with maximum signal fluctuations of up to 37% and 76%, respectively, tracer 1 maintained a very stable *V*_3rms_ signal in all blood concentrations, with a maximum fluctuation of 11%

As seen in Figure 9, all three SPIONs showed a significant signal reduction in 10× and 100× PBS solutions, especially for tracer 2, which was suppressed to near zero under high ion conditions. Since the original calculated Néel relaxation time of each SPION was much faster than the excitation frequency, there was good reason to assume that the MDD interaction and nonspecific binding cannot be ignored. It was important to note that this extremely high concentration of negatively charged ions is not impossible *in vivo*.

Based on the results above, we believe that tracer 1 with a relatively small magnetic core and a thick hydrodynamic diameter is a more ideal clinical tracer for MPI because of its excellent stability under various interference conditions, which is consistent with our theoretical model. However, it was also important to note that the absolute *V*_3rms_ signal strength of tracer 1 was relatively small due to its relatively small core size, which posed a greater challenge to the SNR and sensitivity limit of MPI.

## 4 *In vivo* experiments: methods and results

### 4.1 Narrow-band mixing-frequency theory

As mentioned above, the signal detection sensitivity became challenging when it is necessary to compromise signal strength in exchange for signal stability. Mixing frequency had been widely used as a highly sensitive detection method in magnetic immunology technology. The SPION harmonics under mixing frequency could be modulated to a position where it was different from the excitation frequency or its multiples, which was the largest source of noise in the MPI system.

However, current methods of mixing-frequency detection were not very applicable to MPI because the low-frequency component was only a few tens of hertz, which made the modulated harmonic frequency close to the excitation frequency. Especially in narrow-band MPI, the parallel connected resonant circuit may enhance both the excitation and biased harmonic frequency due to the limitation of the bandwidth and quality factor *Q*.

In this study, a novel mixing-frequency narrow-band MPI detection method was proposed. Here, the excitation field was consisted by a component of high-frequency *f*_1_ with an amplitude *A*_1_ and a component of low-frequency *f*_2_ with an amplitude *A*_2_, and the third mixing-frequency harmonic was generated at *f*_1_ ± 2*f*_2_, which was further enhanced by a narrow-band resonance circuit. Different from previous studies, *f*_1_ was greatly increased to several kHz, which is compatible with the actual bandwidth and *Q* value, while the effective bandwidth of the resonant circuit was always greater than 1 kHz at room temperature, which improved the detection of resonances.

**FIGURE 11:**
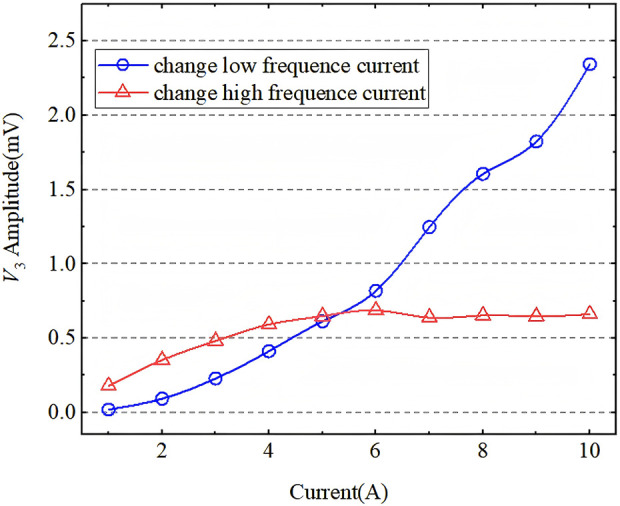
The blue line shows the correlation result of signal strength with *A*_2_, which changed from 1A to 10A, and *A*_1_ was fixed at 5A; the red line shows the correlation result of signal strength with *A*_1_, which changed from 1A to 10A, and *A*_2_ was fixed at 5A.

Figure 11 shows the MPS-measured correlation result of tracer 4 signal strength at *f*_1_ + 2*f*_2_ with different amplitudes of *A*_1_ and *A*_2_. The blue line shows the correlation result of signal strength with *A*_2_, which changed from 1A to 10A, and *A*_1_ was fixed at 5A, while the red line shows the correlation result of signal strength with *A*_1_, which changed from 1A to 10A, and *A*_2_ was fixed at 5A. From the results, we can still obtain large *V*_3rms_ at *f*_1_ + 2*f*_2_ using the proposed method. Interestingly, we found that the strength of *V*_3rms_ increased linearly with *A*_2_ current if *A*_1_ was fixed, while *V*_3rms_ did not increase until *A*_1_ ≈ *A*_2_ if *A*_2_ was fixed. This could be explained by the Taylor expansion of mixing frequency response described by Tu et al. (2013) and Krause et al. (2007):

$$\frac{M}{M_{s}}=L\left(\frac{\mu_{0}mH}{k_{B}T}\right)\approx \frac{1}{3}\left(\frac{\mu_{0}mH}{k_{B}T}\right)-\frac{1}{45}{\left(\frac{\mu_{0}mH}{k_{B}T}\right)}^{3}+\frac{2}{945}{\left(\frac{\mu_{0}mH}{k_{B}T}\right)}^{5}+\cdots +O\left(\frac{\mu_{0}mH}{k_{B}T}\right),$$ (20)

where *H* is a mixing frequency field, *H* = *A*_1_cos(2π*f*_1_ *t*) + *A*_2_cos(2π*f*_2_ *t*); when multiplied out, the third term yields a frequency term of *f*_1_ + 2*f*_2_:

$${\left[A_{1}\cos\left(2\pi f_{1}t\right)+A_{2}\cos\left(2\pi f_{2}t\right)\right]}^{3}=\cdots +\frac{3}{4}A_{1}{A_{2}}^{2}\cos\left[2\pi \left(f_{1}+2f_{2}\right)t\right]+\cdots$$ (21)

The magnetization component at *f*_1_ + 2*f*_2_ could be written as follows:

$$\frac{M_{3}\left(t\right)}{M_{s}}=\frac{A_{1}{A_{2}}^{2}}{60}\cdot \cos\left[2\pi \left(f_{1}+2f_{2}\right)t\right]$$ (22)

The amplitude of the *f*_1_ + 2*f*_2_ term can be expressed as *A*_1_ ·*A*_2_^2^, in which the amplitude of *M*_3_(*t*) is dominated by *A*_2_.

### 4.2 Handheld MPI system

**FIGURE 12:**
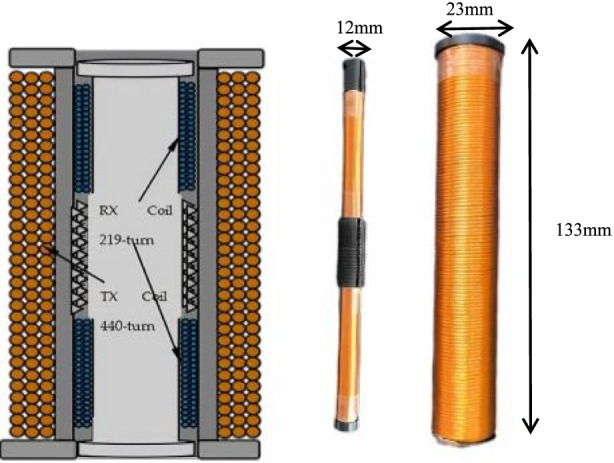
Handheld probe of the MPI system.

The handheld probe of narrow-band mixing-frequency MPI was designed based on the proposed theory. The probe was composed of the outer excitation coil and inner pickup coil in differential structure. The excitation coil was made of 0.1 mm*100 shares Litz wire to reduce the equivalent AC losses with 440 turns and 25 mm in diameter, and the pickup coil is constructed using the same wire in opposite directions at both ends of the detection coil frame, with 219 turns for both the detection and differential parts, enabling mutual cancellation of induced currents generated by the excitation magnetic field, as shown in Figure 12.

**FIGURE 13:**
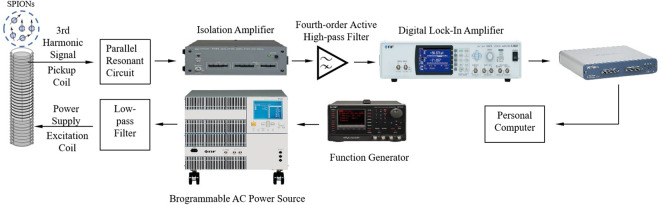
Mixing-frequency narrow-band MPI system.

Figure 13 illustrated the mixing-frequency narrow-band MPI system. A dual-frequency signal was generated synchronously through one channel of the function generator (NI, WF1948), which produced a reference signal to the AC power source (NI, BP4620); another channel yielded a reference signal (*f*_1_ + 2*f*_2_) to the lock-in amplifier (NI, LI5645). The pick-up coil was connected to a self-made parallel resonant circuit (resonance point at 23,270 Hz) to amplify the signal. A self-made active high-pass filter (fourth-order, cutoff frequency of 18 kHz) was used to further remove low-frequency noise from the power supply and other electrical equipment. An isolation amplifier (NI, DC-1 MHz, 5325) was placed between the parallel resonant circuit and the active fourth-order filter to prevent interference between them and also enhance the signal. Then, the higher-order harmonics of nanoparticles was selected by the lock-in amplifier, in which the phase-locked loop further filters out noise signals with different frequencies and phases from the detection signal. The signal was then transmitted to the host computer via a data acquisition card (NI USB6361), with a sampling rate of 2.0 Ms/s.

**FIGURE 14:**
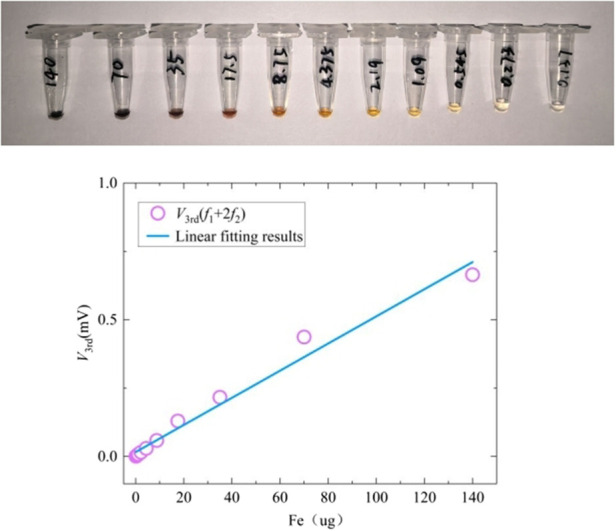
Sensitivity experiment of the handheld MPI sensor. The Resovist sample was diluted into 11 different concentrations, from 0.00274 mg/mL to 28 mg/mL (Fe content range: 0.137 µg–140 µg), all the samples had a volume of 5 µL.

The sensitivity experiment result is presented in Figure 14, which shows the third harmonic response of samples with different concentrations using handheld MPI to verify the performance of the device. Samples were prepared using Resovist and diluted into 11 different concentrations, from 0.00274 mg/mL to 28 mg/mL (Fe content range: 0.137 µg–140 µg). All the samples have a volume of 5 µL. The test parameter was set as follows: *f*_1_ = 15007 Hz, *A*_1_ = 5 A, *f*_2_ = 4123 Hz, *A*_2_ = 5 A, and third harmonic detection frequency was at 23,253 Hz. Signal fluctuation caused by environmental noise and system noise was between 0.0001 and 0.0007 mV. The effective minimum Fe content at 0.273 µg showed an SNR of approximately 8.2 dB; at the Fe content of 140 μg, the SNR reached approximately 59.55 dB. Although signal variations were clearly observable at the Fe content of 0.137 µg(0,0010 mV), it was still too low to be considered the effective sensitivity.

An imaging experiment was also performed by fixing a handheld MPI sensor on a three-axis motion stage to obtain mechanical MPI imaging. A homemade LabVIEW program was used for mechanical control and signal acquisition, while a homemade MATLAB program was used for 2D imaging reconstruction, which was based on the non-negative least squares (NNLS) method. The reconstruction program also supported Z direction tomography if there was only one lymph node at one XY plane, which would be described in detail in later studies.

### 4.3 Sample and animal preparation

Tracers 1, 2, and 4 were used in rabbit SLN detection experiments. All samples were diluted in 4 mg/mL with 1x PBS before injection; the total Fe content was 4 mg in each case. Methylene blue (H32024827, Jumpcan, 0.5 mg/mL) was used as the control, with an injection volume of 1 mL.

All animals used in the experiments were handled in strict accordance with the Animal Ethics Guidelines of China Medical University and approved by the Institutional Animal Care and Use Committee (IACUC) of China Medical University (Approval No. 2025G0774). All animals were housed under standard laboratory conditions and allowed a 1-week acclimatization period prior to the experiments. Prior to anesthesia induction, the body weight of each animal was accurately measured using an electronic balance (precision: 0.01 g) for the precise calculation of anesthetic dosage. One percent of the sodium pentobarbital solution was used as both anesthetic and euthanasia agents. Anesthesia was initially induced via intravenous injection through the auricular vein at a dose of 50 mg/kg body weight. The experiment was initiated only after the confirmation of a deep anesthetic state, which was defined by the loss of righting reflex, diminished corneal reflex, and the absence of the withdrawal response to toe pinch stimulation. Upon completion of the experiment, it was confirmed that the animals remained in a deep anesthetic state, and humane euthanasia was performed promptly via the same route with an overdose of 1% sodium pentobarbital solution at 150 mg/kg body weight. The euthanasia process was continuously monitored, and animal death was verified using two independent methods: ① permanent cessation of spontaneous thoracic respiration sustained for at least 5 min; ② absence of heartbeat confirmed by palpation of the left thoracic region. If necessary, thoracotomy was performed to directly observe the static state of the heart, thereby preventing pseudodeath.

### 4.4 Animal experiment

**FIGURE 15:**
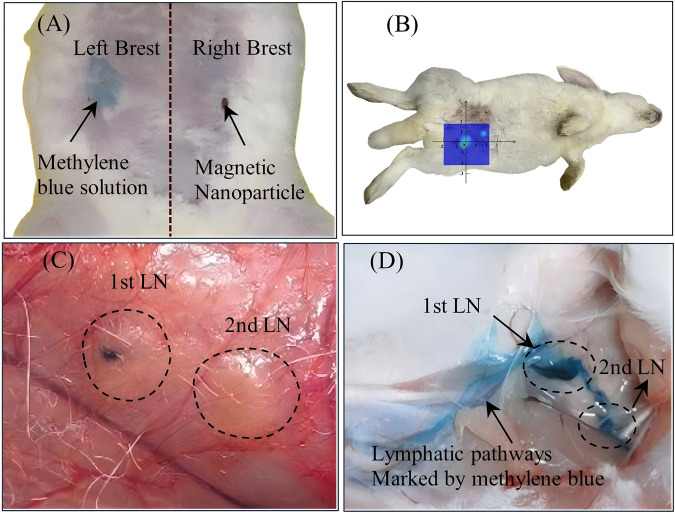
**(A)** Methylene blue injected in the left breast, and SPIONs injected in the right breast; **(B)** lymph node imaging results of MPI on the body surface of rabbits labeled by tracer 1; **(C)** two SPION-labeled lymph nodes observed in the mammary region of rabbits through dissection; **(D)** two methylene blue-labeled lymph nodes observed in the mammary region of rabbits through dissection.

First, we conducted an SLN intraoperative localization experiment. After anesthetization, 1 mL each from three tracers and methylene blue solution were injected subcutaneously into the breast area near the second nipple of two rabbits (Figure 15A) and then massaged for 5 min. It was well known that the outer diameter of SPIONs from 60 nm to 150 nm can penetrate the lymphatic system and aggregate in SLN (Natalie et al., 2015), while methylene blue, due to its small molecular weight, may simultaneously penetrate blood vessels or other nearby lymph nodes. In the MPI group, the first lymph node was dyed brown, which could be recognized by both the naked eye and MPI, and the second lymph node was difficult to identify by the naked eye, while it could be found using the MPI signal (Figures 15C,D). The MPI signal of the first lymph node was much larger than that of the second node, which had been proven to be the SLN. The methylene blue group could also identify two lymph nodes, but it was difficult to separate the SLN on the basis of color, and they had also spread to other tissues such as nearby capillaries, causing a certain degree of intraoperative contamination. Figure 15B shows the concentration distribution result of the rabbit mammary gland region. We achieved significant MPI highlights at the injection point and LN1, but LN2 is barely visible in the image. This is mainly because the handheld MPI did not have a gradient field to achieve millimeter-level spatial resolution; LN1 and LN2 were not effectively distinguished by imaging. However, in any case, since LN2 is not generally considered SLN in the medical field, we can assume that the currently developed MPI systems and SPIONs can achieve *in vivo* SLN imaging, which is very important to improve the efficiency and accuracy of breast cancer surgery.

**FIGURE 16:**
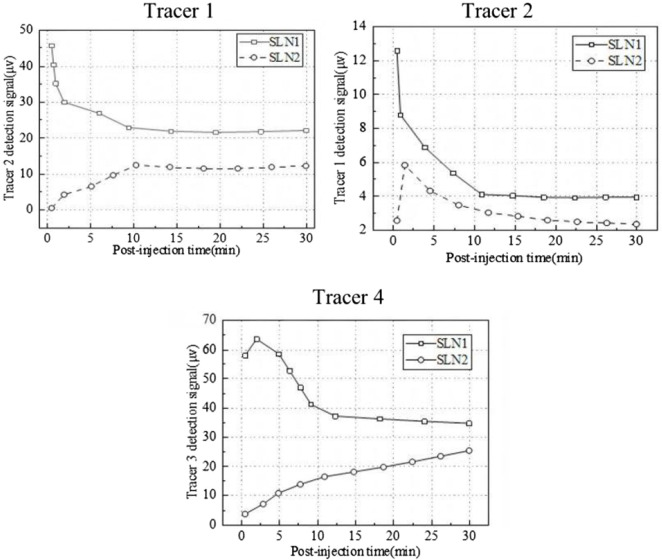
Continuous signal of three tracers from 0 to 30 min after message and cutting open.

Figure 16 shows the continuous signal of three tracers from 0 to 30 min after cutting open. It can be observed that both lymph nodes showed a relatively stable signal from 10 to 30 min in the case of tracer 1, which can be considered the optimal detection time window, while for tracer 2, it continued to decrease within 30 min. It is important to note that the absolute value of tracer 2 is much smaller than that of tracer 1, suggesting that it is severely affected by the *in vivo* environment. Moreover, the signal of tracer 1 was maintained at a very stable value even at 30 min; therefore, we thought that the effective time window was much longer, which should be confirmed in further studies.

In the case of tracer 4, the measured signal of the first lymph node kept decreasing while increasing in the second lymph node, and both signals remained unstable for 30 min. The instability of signals was due to either biological or physical reasons as mentioned above, but the specific reasons here are not easy to analyze due to the lack of a contrast tracer with the same coating. However, it is obvious that the absolute signal intensity of tracer 4 was in the same range as that of tracer 1 with the same Fe concentration, which was very different from that in Figure 10, indicating that tracer 4 was seriously affected by *in vivo* interference.

**FIGURE 17:**
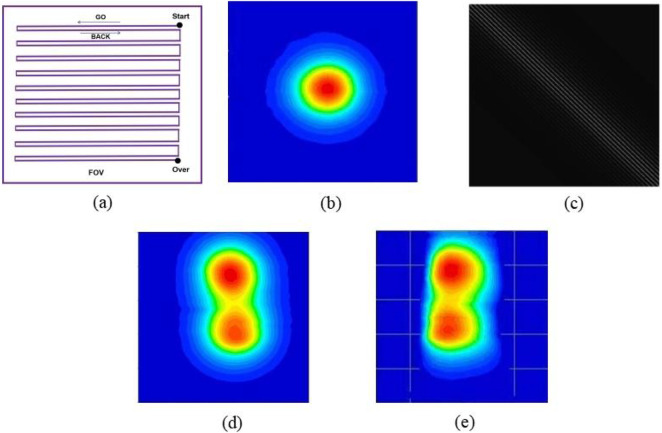
**(a)** Schematic diagram of the robotic arm movement trajectory; **(b)** schematic diagram of the PSF obtained by scanning standard sample points; **(c)**the system matrix constructed from the PSF; **(d)** voltage results before reconstruction; **(e)** concentration results after reconstruction.

We further performed MPI imaging experiments with Tracer 1 within 30 min after suturing the rabbits. The imaging area was set to 4 cm × 4 cm, and the moving step size of the robotic arm driving the probe was 1 mm; the robotic arm movement trajectory is shown in Figure 17a. It should be especially noted that since no gradient magnetic field was used in the imaging process of this study, the image resolution depends entirely on the moving step size of the robotic arm and the full width at half maximum (FWHM) of the nanoparticles.

Generally, the raw images acquired by MPI are voltage maps. However, for clinical applications, the local concentration of the tracer is more meaningful than the raw voltage signal in tracer imaging. Therefore, we reconstructed the measured voltage signals to obtain the concentration distribution of the MPI image. The voltage signals collected point-by-point by the sensor form the voltage matrix *V*_*mn*_, as shown in Equation 24. As a tracer imaging technique, MPI relies more on tracer concentration to provide clinically relevant information. For this reason, we usually convert the voltage matrix into a concentration matrix using mathematical methods. The relationship between voltage and concentration can be simply expressed as follows: A*c_*mn*_ = *v*_*ij*_, where A is the system matrix, which describes the quantitative mapping between the sensor signals and the physical distribution within the region to be reconstructed; *c*_*mn*_ is the sample concentration, as shown in Equation 23; and *V*_*mn*_ is the voltage matrix, as presented in Equation 24.

$$c_{ij}=\left(\begin{array}{cccc} c_{11} & c_{12} & \ldots & c_{1j}\\ c_{21} & c_{22} & \ldots & c_{2j}\\ \vdots & \vdots & \ddots & \vdots \\ c_{i1} & c_{i2} & \ldots & c_{ij} \end{array}\right),$$ (23)

$$V_{mn}=\left(\begin{array}{cccc} V_{11} & V_{12} & \ldots & V_{1n}\\ V_{21} & V_{22} & \ldots & V_{2n}\\ \vdots & \vdots & \ddots & \vdots \\ V_{m1} & V_{m2} & \ldots & V_{mn} \end{array}\right),$$ (24)

$$V_{\text{mn}}=\sum a_{mn,ij}c_{ij}.$$ (25)

Equation 25 defines the relationship between the voltage and concentration for each signal point, where *a*_*mnij*_ represents an element of the system matrix *A*. The ordered arrangement of all *a*_*mnij*_ elements constitutes the system matrix *A* (as shown in Equation 26). In general, the system matrix *A* is constructed by the regular arrangement of experimentally measured point spread functions (PSFs).

$$A=\left(\begin{array}{ccccccc} a_{1111} & a_{1112} & \ldots & {\;a}_{11\left(1j\right)} & {\;a}_{1121} & \ldots & a_{11\left(ij\right)}\\ a_{1211} & a_{1212} & \ldots & a_{12\left(1j\right)} & {\;a}_{1121} & \ldots & a_{12\left(ij\right)}\\ \vdots & \vdots & \ddots & \vdots & \vdots & \ddots & \vdots \\ a_{\left(1n\right)11} & a_{\left(1m\right)12} & \ldots & a_{\left(1n\right)\left(1j\right)} & a_{\left(1n\right)21} & \ldots & a_{\left(1n\right)\left(ij\right)}\\ a_{2111} & a_{2112} & \ldots & a_{21\left(1j\right)} & a_{2121} & \ldots & a_{2n\left(ij\right)}\\ \vdots & \vdots & \ddots & \vdots & \vdots & \ddots & \vdots \\ a_{\left(mn\right)11} & a_{\left(mn\right)12} & \ldots & a_{\left(mn\right)\left(1j\right)} & {\;a}_{\left(mn\right)21} & \ldots & a_{\left(mn\right)\left(ij\right)} \end{array}\right).$$ (26)

The PSF refers to the scanning data from a standard sample with a predefined spatial position and concentration (e.g., a high-concentration sample placed at the central position) acquired using the MPI system, as shown in Figure 17b. It is used to characterize the correlation between the imaging results of the MPI system and the spatial position, as well as the concentration of the sample. The system matrix *A* required for MPI image reconstruction is generated from the PSF. In this study, the PSF was acquired by scanning a standard sample spot (using the same tracer as that of the sample to be reconstructed) under identical measurement conditions—where the measurement conditions refer to the exact same measurement parameters adopted for scanning the sample to be reconstructed, including the same excitation current, detection distance, sampling rate, and scanning area. The system matrix *A* was then constructed by simulating the distribution of scanning results of the standard sample spot at each position within the scanning region based on the PSF matrix, as shown in Figure 17c. To verify the system's spatial resolution, we performed scanning tests using two circular samples with a diameter of 5 mm. The results show that the two samples can be clearly distinguished when the distance between the two samples is 7 mm in the absence of a gradient magnetic field. When the distance between the two samples is less than 7 mm, the central red regions of the samples begin to overlap. Meanwhile, we adopted the system matrix method and restored the collected voltage signals to the nanoparticle concentration values in the target area through the NNLS algorithm, realizing the accurate reconstruction of concentration images. Figures 17d,e show the voltage results before reconstruction and concentration results after reconstruction, respectively. Since no gradient magnetic field was applied during scanning, the reconstruction process hardly contributed to the resolution; it only converted the voltage results back to concentration results.

## 5 Discussion

This study focused on the stability and quantifiability of SPION carriers under the MPI detection system in complex *in vivo* interference conditions. By assuming that SPIONs are impossible to ensure their stability under interference factors such as negative ions, protein unspecific binding, and cell phagocytosis, the aggregated or immobilized SPIONs must maintain their AC harmonic response in a relatively stable state.

One of the main contributions of this study was the summary of the biological factors and physics principles that may affect the stability of SPIONs and its AC response signal. We found that Néel-dominated SPIONs smaller than 30 nm could also be affected due to the MMD interference or easy axis rearrangement, which could be a key factor affecting the quantitative accuracy.

We, therefore, established a theoretical model for the development and evaluation of high-signal-stability SPIONs. A stability evaluation is given in Equation 16, with the analysis of signal strength losses (Equations 17–19), so that the developers could comprehensively consider the stability and signal strength of SPIONs and the development of the MPI system. Based on the study on ACS, we found that the specially designed effective value was the key for low-fluctuation harmonic signal measurement, although some compromise of single-domain magnetic moments and the thickness of hydrodynamic coating is necessary, both of which cause the loss of absolute signal strength as well. The newly developed SPIONs (tracer 1) showed excellent harmonic magnetization stability under different interferences, including high ions, high viscosity, serum, and whole-blood environments.

A novel narrow-band mixing-frequency theory was proposed to compensate for the signal loss. Different from previous studies, the narrow-band mixing-frequency theory requires that both components have a large frequency and intensity. The new signal acquisition method significantly contributes to SNR and high-*Q* resonance, which enhances signal strength compared to the traditional mixing-frequency theory. A handheld mixing-frequency MPI system was then developed for SLN detection in rabbits, which exhibited excellent sensitivity and verified the *in vivo* stability of 20-nm SPION carriers.

## 6 Conclusion

In this study, we established a stable theoretical model for SPION carriers that enables quantitative detection and conducted both *in vitro* and *in vivo* validation experiments on SPIONs with different sizes; the results were in good agreement with the theoretical predictions derived from the model. Furthermore, to address the issue of weak magnetization signals of SPIONs under the stable model, we proposed a novel narrow-band mixing-frequency theory for the MPI detection system, which is designed to compensate for the signal attenuation of highly stable carriers. The efficacy of the proposed theory and associated technology was verified via rabbit SLN detection and imaging experiments. We anticipate that the developed theoretical framework, SPIONs, and MPI system will be translated into clinical applications in the future.

## Data Availability

The original contributions presented in the study are included in the article/supplementary material; further inquiries can be directed to the corresponding authors.
